# Effects of Organic Fertilizer on Photosynthesis, Yield, and Quality of Pakchoi under Different Irrigation Conditions

**DOI:** 10.3390/plants13101308

**Published:** 2024-05-09

**Authors:** Shudong Lin, Kai Wei, Quanjiu Wang, Yan Sun, Mingjiang Deng, Wanghai Tao

**Affiliations:** State Key Laboratory of Eco-Hydraulics in Northwest Arid Region, Xi’an University of Technology, Xi’an 710048, China; shudong_lin@163.com (S.L.);

**Keywords:** organic fertilizer, photosynthetic characteristics, light response model, pakchoi, yield, quality

## Abstract

Water scarcity and the overuse of chemical fertilizers present significant challenges to modern agriculture, critically affecting crop photosynthesis, yield, quality, and productivity sustainability. This research assesses the impact of organic fertilizer on the photosynthetic attributes, yield, and quality of pakchoi under varying irrigation water conditions, including fresh water and brackish water. Findings reveal that the modified rectangular hyperbolic model most accurately captures the photosynthetic reaction to organic fertilization, outperforming other evaluated models. The maximum net photosynthesis rate (P_nmax_), yield, soluble sugar (SS), and soluble protein content (SP) all exhibited a downward-opening quadratic parabolic trend with increasing amounts of organic fertilizer application. Specifically, under fresh-water irrigation, the optimal P_nmax_, yield, SS, and SP were obtained at organic fertilizer rates of 65.77, 74.63, 45.33, and 40.79 kg/ha, respectively, achieving peak values of 20.71 µmol/(m^2^·s), 50,832 kg/ha, 35.63 g/kg, and 6.25 g/kg. This investigation provides a foundational basis for further research into the intricate relationship between water salinity stress and nutrient management, with the goal of crafting more sophisticated and sustainable farming methodologies. The insights gained could significantly influence organic fertilizer practices, promoting not only higher yields but also superior quality in agricultural outputs.

## 1. Introduction

In arid regions, where water resources are scarce, enhancing agricultural production efficiency is crucial for economic growth [1]. Concurrently, soil salinization poses a global challenge in dry and semi-arid zones, reducing plant diversity and inhibiting soil microbial activity, which can lead to land desertification and water pollution [2].

Arid areas commonly experience a dearth of fresh water, often exacerbated by its over-extraction for industrial and agricultural use. This is notably evident in the extensive Southern Xinjiang province of China, where fresh water is limited and evaporation rates are high [3,4]. Such water scarcity hampers crop growth and development, diminishing photosynthetic efficiency and thereby impacting yields and quality. Insufficient water availability curtails the crop root system water absorption, which is essential for photosynthesis, adversely affecting plant growth. Sensible use of brackish water serves as a viable solution to mitigate local water deficits, diversify agricultural water supplies, and counteract drought [5]. Nonetheless, the elevated mineral content in brackish water used for irrigation can escalate soil salinity, which hinders crop vitality and degrades soil quality [6,7]. Moreover, using brackish water for irrigation can precipitate physiological drought, necessitating adaptive physiological responses in crops and influencing the allocation of photosynthates [8].

Building upon traditional practices of water and nutrient management, the use of chemical fertilizers in agriculture can indeed initially elevate crop yields [9]; however, this short-term gain is overshadowed by long-term consequences, such as the degradation of soil structure, decline in fertility, and environmental pollution, which collectively compromise crop quality and the sustainable future of agriculture. Excessive application of elements like nitrogen, phosphorus, and potassium not only squanders resources but also contributes to the buildup of soil salinity, impairing both soil aeration and moisture retention [10,11,12]. Over time, these conditions can inflict significant damage on soil health and diminish its productivity. Conversely, organic fertilizers, which are abundant in organic materials, microbial life, and a mix of organic and inorganic nutrients, offer multifaceted benefits for improving the physical, chemical, and biological attributes of soil [7,13,14]. Research consistently shows that sensible utilization of organic fertilizers can substantially bolster crop photosynthesis, leading to increased yields across a diversity of crops [15,16,17]. Furthermore, organic fertilizers can improve crop quality by enhancing levels of soluble sugar, vitamin C, and organic matter while also refining the growth environment and the soil mineral profile [18,19]. Strategically combining organic with inorganic fertilizers in various proportions has also been shown to positively influence crop growth [20,21].

Pakchoi, as a member of the genus Brassica in the Cruciferae family, also referred to as oilseed rape or green vegetable, is favored for its crisp and tender edible stems and leaves [22]. Its widespread appeal is due to its richness in essential minerals and vitamins. The yield of pakchoi is predominantly influenced by water fertilizer management and photosynthesis [23]. The light response curve is an effective assessment tool for delineating the variability in the net photosynthesis rate under different intensities of photosynthetically active radiation [24]. Researchers utilize various light response models (including the rectangular hyperbolic, non-rectangular hyperbolic, exponential, and modified rectangular hyperbolic models) to extract key physiological parameters and, thus, to enhance the understanding of the photosynthetic mechanisms of the crop [25]. Currently, comprehensive insights into the light response curves of pakchoi are essential, as they are intrinsically linked to the plant biological traits and ecological adaptability, offering vital data for plant breeding and genetic advancements.

Scholars globally have extensively studied the light response mechanisms of staple crops such as wheat, corn, and rice [26,27,28]. Nonetheless, studies on the influence of organic fertilizer coupled with irrigation using fresh water and brackish water on the light response traits of pakchoi, as well as the suitability of various light response models, remain sparse. This study hypothesized that organic fertilizer may bolster soil structure and environment conditions, augmenting the plant capacity for water and nutrient uptake, consequently enhancing photosynthesis, yield, and quality. The study aims are (1) to clarify the effects of different organic fertilizer application rates on pakchoi light response; (2) to pinpoint the most fitting light response model and parameters for pakchoi; and (3) to establish the optimal rate of organic fertilizer for pakchoi. The overarching goal of this research is to lessen the dependence on synthetic fertilizers, encourage the employment of organic and biological fertilizers, and boost fertilizer use efficiency, thereby advancing crop photosynthesis, yield, and quality in pursuit of eco-friendly and sustainable agriculture.

## 2. Materials and Methods

### 2.1. Experimental Site and Description

**Figure 1:**
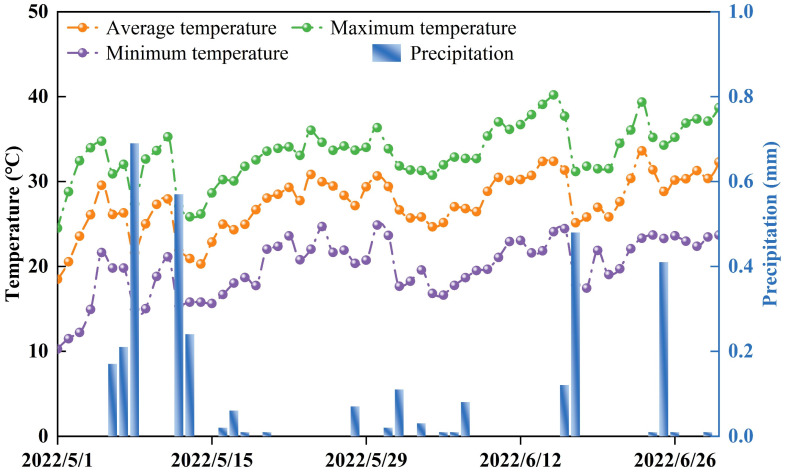
Temperature and precipitation during the whole growth period of pakchoi.

The study was conducted at the Bazhou Irrigation Experiment Station in Korla City, Xinjiang, China. The station, located at coordinates 41°45′20.24″ N, 86°8′51.16″ E, is situated at an altitude of 901 m. This locale was emblematic of the warm temperate zone and is distinguished by its continental arid climate. The study was completed in 2022, meticulously considering the unique meteorological patterns of the region. The site boasts a frost-free duration, ranging from 175 to 200 days per annum, and substantial solar exposure, receiving between 1794 and 1847 h of sunlight annually. Table 1 documents the initial soil assessments conducted in preparation for the research, detailing the physical and chemical attributes of the soil. Meteorological data (Figure 1), including daily average temperature, maximum and minimum temperatures, and precipitation levels, were collected using an automated meteorological unit (Davis VantagePro-6152) on-site.

### 2.2. Experimental Design and Field Management

The non-heading variant of pakchoi, noted for its rapid vegetative growth, brief life cycle, and nutrient density across all seasons, was chosen as the subject of this investigation. The customary irrigation quota, established at approximately 450 m³/ha, was derived from the annual hydration demands for pakchoi cultivation specific to this region. The study employed a water-soluble bio-organic fertilizer (Shidiji biological organic fertilizer, Chengdu Huahong Biotechnology Co., Ltd., Chengdu, China) comprising a minimum of 40% organic matter, 5% N, P_2_O_5_, and K_2_O, 10% amino acids, and 5% fulvic acid. This fertilizer was administered through the irrigation water at eight distinct intervals during the plant growth period. The experimental design incorporated a flood irrigation methodology featuring five levels of bio-organic fertilizer amounts (0, 20, 40, 60, and 80 kg/ha) in conjunction with two water variants, which were fresh water (F) and brackish water (B). The treatments were labeled as follows: F0, F1, F2, F3, and F4 for the F treatments; and B0, B1, B2, B3, and B4 for the B treatments, corresponding to the respective fertilizer increments.

Sowing of the seeds occurred on the 7 May 2022, with the respective harvests taking place on the 30 June. To ensure experimental integrity, each plot was delineated with a high-density, impermeable membrane extending to a depth of 2 m. The fertilizer treatment plots were maintained consistently over a two-year period and were subjected to three replications within a randomized block design framework. Each plot was 3 m^2^ in area, with a seeding density of 2.5 × 10^5^ plants/ha. Cultivating crops, including removing weeds and implementing measures to control pests and diseases, adhered to the established agricultural protocols specific to the local area.

### 2.3. Measurements and Calculations

#### 2.3.1. Measurements of Pakchoi Samples

The yield of pakchoi at maturity is quantified based on its fresh weight. Yield calculation, as per Equation (1), involves harvesting and weighing all pakchoi plants from each plot [29]. The soluble sugar (SS, g/kg) and soluble protein (SP, g/kg) content in pakchoi were measured using the anthrone and Coomassie Brilliant Blue UV spectrophotometry methods, respectively (Shimadzu, Kyoto, Japan, UV-1900i) [30,31].

(1) $$Yield=FW_{p}\times P$$

where *FW_p_* is the average fresh weight of per plants (kg/plant), *P* represents the plant density of pakchoi (plants/ha).

To determine the light response parameters of pakchoi, this study selects the most vigorous growth period of pakchoi. Three pakchoi leaves exhibiting robust growth and full expansion at their upper sections are randomly selected between 09:00 and 11:00 on a sunny day. Using a Li-6400 portable photosynthesis system equipped with a red−blue light source, photosynthetically active radiation levels are set at the following 15 gradients: 2500, 2200, 2000, 1800, 1600, 1400, 1200, 1000, 800, 600, 400, 200, 100, 50, and 0 µmol/(m^2^·s). This setup enables the measurement of the net photosynthesis rate, P_n_ (µmol/(m^2^·s)), transpiration rate (T_r_, mmol/(m^2^·s)), stomatal conductance (G_s_, mol/(m^2^·s)), intercellular CO_2_ concentration (C_i_, µmol/mol), and other vital parameters of pakchoi, from which a light response curve is subsequently plotted.

#### 2.3.2. Light Response Curve Model

Net photosynthesis rate (P_n_) serves as a crucial metric for assessing plant health and productivity, signifying the plant capacity to synthesize organic compounds from atmospheric CO_2_, which is essential for growth.

(1) Rectangular hyperbolic model [32]:

(2) $$P_{n}=\frac{\alpha IP_{n\max}}{\alpha I+P_{n\max}}-R_{d}$$

where P_n_ is the net photosynthesis rate, µmol/(m^2^·s); α is the apparent quantum efficiency, also known as the initial quantum efficiency, which is the initial slope of the light response curve (I = 0) and can reflect the ability of crops to absorb, convert, and utilize light energy under low light; P_nmax_ is the maximum net photosynthesis rate, µmol/(m^2^·s), which can reflect the maximum photosynthetic capacity of leaves; R_d_ is the dark respiration rate, µmol/(m^2^·s), which can reflect the respiration rate of crop leaves under no light conditions; and I was photosynthetically active radiation, µmol/(m^2^·s).

The light compensation point (I_c_, µmol/(m^2^·s)) is calculated by the following formula. The lower the I_c_ value, the stronger the ability of crops to utilize low light intensity. The calculation formula is as follows:

(3) $$I_{c}=\frac{P_{n\max}R_{d}}{\alpha \left(P_{n\max}-R_{d}\right)}$$

Equation (3) is a function without extreme value, which reflects that the right-angle hyperbola is an asymptotic trend line that cannot reach the pole; that is, the light saturation point (I_sat_, µmol/(m^2^·s)) cannot be directly calculated by Equation (2). The greater the light saturation point, I_sat_, the less likely it is to have photoinhibition under the stimulation of strong light in the process of crop growth and development.

The line $y=P_{n\max}$ intersects with the linear equation line $y=\alpha I-R_{d}$ under low light (I ≤ 200 µmol/(m^2^·s)), and the I value corresponding to the x-axis corresponding to the intersection point is the I_sat_.

(2) Non-rectangular hyperbolic model [32]:

(4) $$P_{n}=\frac{\alpha I+P_{n\max}-\sqrt{{\left(\alpha I+P_{n\max}\right)}^{2}-4k\alpha IP_{n\max}}}{2k}-R_{d}$$

where *k* is the angle of non-rectangular hyperbolic parameter, 0 < *k* ≤ 1. If the model fits well, the following formula can be used to calculate the *I_c_*:

(5) $$I_{c}=\frac{P_{n\max}R_{d}-k{R_{d}}^{2}}{\alpha \left(P_{n\max}-R_{d}\right)}$$

(3) Modified rectangular hyperbolic model [33]:

(6) $$P_{n}=\alpha \frac{1-\beta I}{1+\gamma I}I-R_{d}$$

where β is the photoinhibition coefficient, and γ is a coefficient independent of I. P_nmax_, I_c_ and I_sat_ are calculated from Equations (7)–(9).

(7) $$P_{n\max}=\alpha {\left(\frac{\sqrt{\beta +\gamma }-\sqrt{\beta }}{\gamma }\right)}^{2}-R_{d}$$

(8) $$I_{sat}=\frac{\sqrt{\left(\beta +\gamma \right)/\beta }-1}{\gamma }$$

(9) $$I_{c}=\frac{\alpha -\gamma R_{d}-\sqrt{{\left(\gamma R_{d}-\alpha \right)}^{2}-4\alpha \beta R_{d}}}{2\alpha \beta }$$

(4) Exponential model [34]:

(10) $$P_{n}=P_{n\max}\left(1-e^{-\alpha I/P_{n\max}}\right)-R_{d}$$

When estimating I_sat_, it is assumed that the light intensity corresponds to P_n_ of 0.99 × P_nmax_ and is saturated light intensity. I_c_ and I_sat_ are calculated from Equations (11) and (12).

(11) $$I_{sat}=-\frac{P_{n\max}\ln\left(0.1\right)}{\alpha }$$

(12) $$I_{c}=\left(-\frac{P_{n\max}}{\alpha }\right)\ln\left(1-\frac{R_{d}}{P_{n\max}}\right)$$

### 2.4. Statistical Analysis

Microsoft Office Excel (2016)(Microsoft Corporation, Redmond, WA, USA) and Origin 2021 (OriginLab, Northampton, MA, USA) were used for data processing and graphical representation, respectively. One-way analysis of variance (ANOVA) was performed using SPSS 25.0 (2017, IBM, Crop., Armonk, NY, USA) with a significance threshold set at *p* < 0.05 to determine statistically significant differences. The establishment and resolution of the model were accomplished through MATLAB (2014a)(MathWorks Inc., Natick, MA, USA) programming. Concurrently, the determination of correlations employed the coefficient of determination (*R*^2^), while the accuracy assessment was conducted using root mean square error (RMSE) and relative error (RE).

## 3. Results

### 3.1. Photosynthetic Physiological Characteristics

**Figure 2:**
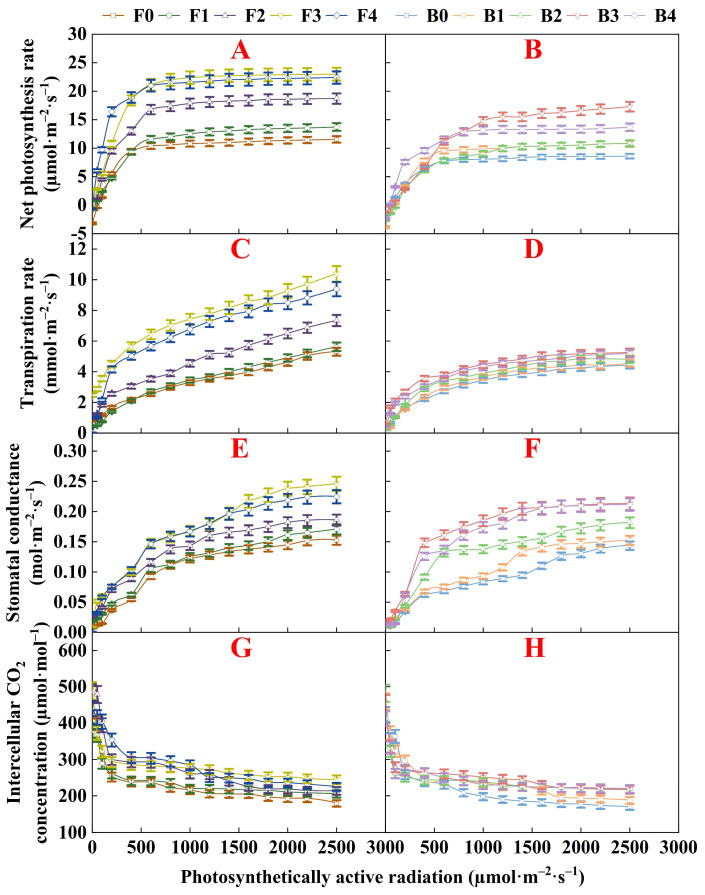
Net photosynthesis rate (**A**,**B**), transpiration rate (**C**,**D**), stomatal conductance (**E**,**F**), and intercellular CO_2_ concentration (**G**,**H**) characteristics of pakchoi under fresh water and brackish water irrigation. F0, F1, F2, F3, and F4 denote the application rates of organic fertilizer at 0, 20, 40, 60, and 80 kg/ha, respectively, under fresh-water irrigation. B0, B1, B2, B3, and B4 denote the application rates of organic fertilizer at 0, 20, 40, 60, and 80 kg/ha, respectively, under brackish water irrigation.

This study delineates the dynamics of P_n_, T_r_, G_s_, and C_i_, as they respond to I under irrigation with both fresh water and brackish water in pakchoi (Figure 2A–H). Notably, P_n_ exhibits a rapid increase with I values below 400 µmol/(m^2^·s). However, upon exceeding 1000 µmol/(m^2^·s), the increase in P_n_ decelerates, approaching a light saturation point indicative of the peak net photosynthesis rate. Observations within a stable light intensity range reveal a descending order of organic fertilizer application correlating with P_n_ as follows: 60 kg/ha, 80 kg/ha, 40 kg/ha, 20 kg/ha, and 0 kg/ha.

Figure 2 present the response of T_r_ and G_s_ to I under various treatments in pakchoi. Across all treatments, T_r_ and G_s_ exhibit a swift increase as I rise. Beyond a I of 400 µmol/(m^2^·s), the rates of increase for both T_r_ and G_s_ decelerate. Similarly, the behavior of C_i_ with respect to I shifts demonstrates a notable reduction in C_i_ at low light intensities (I < 400 µmol/(m^2^·s)). With escalating light intensity (400 µmol/(m^2^·s) < I < 2500 µmol/(m^2^·s)), C_i_ gradually declines across treatments until it reaches a steady state. This trend is likely due to the rapid surge in leaf P_n_ and subsequent elevated CO_2_ consumption under initially low light conditions, resulting in a significant drop in C_i_. Once I surpass a specific limit, the acceleration of photosynthesis diminishes, leading to more gradual modifications in C_i_.

Overall, the effects of fresh water and brackish water irrigation on photosynthetic parameters exhibit similar patterns, indicating that these two irrigation methods have consistent effects on plant photosynthetic characteristics and water use efficiency. However, photosynthetic parameters show a clear advantage under fresh-water irrigation, especially in P_n_, T_r_ and G_s_.

### 3.2. Applicability Evaluation of Light Response Model

**Figure 3:**
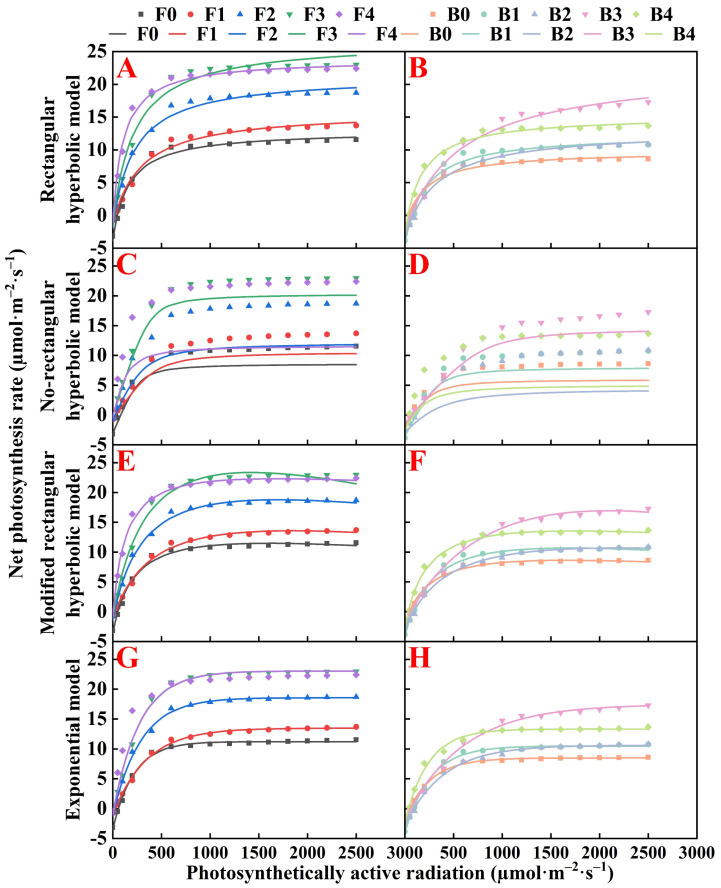
The fitting effect of four models on the light response curve under fresh water and brackish water irrigation. Rectangular hyperbolic model (**A**,**B**), Non-rectangular hyperbolic model (**C**,**D**), Modified rectangular hyperbolic model (**E**,**F**), Exponential model (**G**,**H**). F0, F1, F2, F3, and F4 denote the application rates of organic fertilizer at 0, 20, 40, 60, and 80 kg/ha, respectively, under fresh-water irrigation. B0, B1, B2, B3, and B4 denote the application rates of organic fertilizer at 0, 20, 40, 60, and 80 kg/ha, respectively, under brackish water irrigation. Solid points represent measured values and solid lines represent fitted values.

This study applied four light response models to characterize the light response curves of pakchoi under both fresh water and brackish water irrigation; these models included the rectangular hyperbola, non-rectangular hyperbola, exponential, and modified rectangular hyperbola models (Figure 3A–H). The first three models failed to effectively capture the variations in P_n_ past the light saturation point due to their lack of extreme value asymptotes. In contrast, the modified rectangular hyperbola model precisely matched the light response curves, particularly those displaying photoinhibition. Nevertheless, the observed light intensity range in this study did not show a declining P_n_ trend for pakchoi under any treatment, indicating an absence of photoinhibition. Comparative analysis of the models, as depicted in Figure 3 and Table 2, revealed that the non-rectangular hyperbola model performed the least effectively. The rectangular hyperbola, modified rectangular hyperbola, and exponential models all adequately described the light response of pakchoi leaves. Among them, the modified rectangular hyperbola model demonstrated the closest alignment with the actual data trends, showcasing superior fitting accuracy (*R*^2^ ≥ 0.99 and the lowest RMSE and RE) compared to the other models. This finding positions the modified rectangular hyperbola model as the most accurate and optimal choice for depicting the light response curves of pakchoi leaves across different organic fertilizer treatments.

### 3.3. Change Characteristics of Light Response Model Parameters

To quantitatively assess the impact of various treatments on pakchoi light response, parameters from the modified rectangular hyperbola model, such as α, P_nmax_, I_c_, I_sat_, and R_d_ were determined. Plants exhibiting a lower I_c_ and higher I_sat_ demonstrate superior adaptability to light, efficiently utilizing low light and thriving in intense light conditions. In contrast, plants with a higher I_c_ and lower I_sat_ show reduced adaptability to light variations. Therefore, the range of usable light intensity for pakchoi leaves is represented by I_sat_ − I_c_ (∆I, µmol·m^−2^·s^−1^) (Table 3).

**Figure 4:**
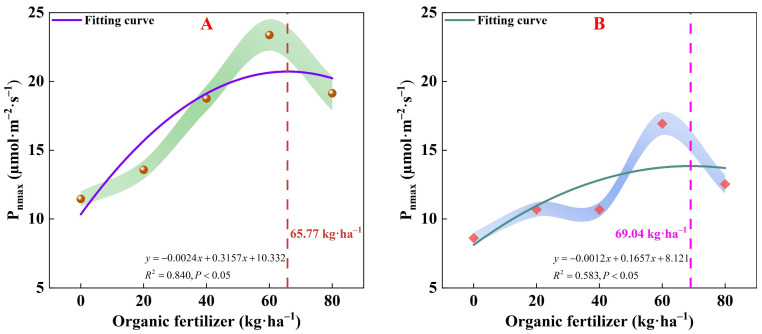
Relationship between maximum net photosynthesis rate and organic fertilizer under fresh water (**A**) and brackish water (**B**) irrigation. P_nmax_, maximum net photosynthesis rate.

Distinct differences in α, P_nmax_, I_c_, I_sat_, and R_d_ were noted across various organic fertilizer treatments. Specifically, treatments F4 and B4 showed a notably higher α value and a lower I_c_, indicating enhanced low light utilization and shade tolerance, facilitating normal growth under diminished light intensity. The I_sat_ and ∆I value were considerably higher in treatments F1 and B2, indicating that pakchoi photosynthesis was less susceptible to inhibition by strong light, thus improving its resilience to high light levels and adaptability to light conditions. A higher R_d_ reflects increased leaf physiological activity and accelerated consumption of organic and nutritional substances within the plant. The lowest R_d_ observed in treatments F3 and B3 minimized respiratory consumption, allowing for organic accumulation and adaptation to environmental stress. Under fresh-water irrigation, the application of organic fertilizer more effectively improved the P_nmax_ of pakchoi. The use of organic fertilizer increased the range of light intensity usable by pakchoi under brackish water irrigation, but it also accelerated the consumption of organic matter and nutrients during the growth process of pakchoi. As fertilizer amounts increased, P_nmax_ initially rose and then fell, reaching its peak under treatments F3 and B3. Under fresh-water irrigation, when the organic fertilizer application rate is 65.77 kg/ha, the P_nmax_ reaches its peak at 20.71 µmol/(m^2^·s) (Figure 4A). Under brackish water irrigation, with an organic fertilizer application rate of 69.04 kg/ha, P_nmax_ attains its maximum value of 13.84 µmol/(m^2^·s) (Figure 4B).

This analysis suggests that organic fertilizer application can significantly enhance pakchoi photosynthesis, especially under brackish water irrigation. Applying 80 kg/ha of organic fertilizer improves low light utilization, whereas 20–40 kg/ha enhances strong light utilization, albeit with higher R_d_ values. When the application rate of organic fertilizer is 60 kg/ha, it not only enhances the efficiency of pakchoi in utilizing low light but also facilitates the accumulation of biomass.

### 3.4. Relationship between Photosynthetic Characteristics and Yield Quality

**Figure 5:**
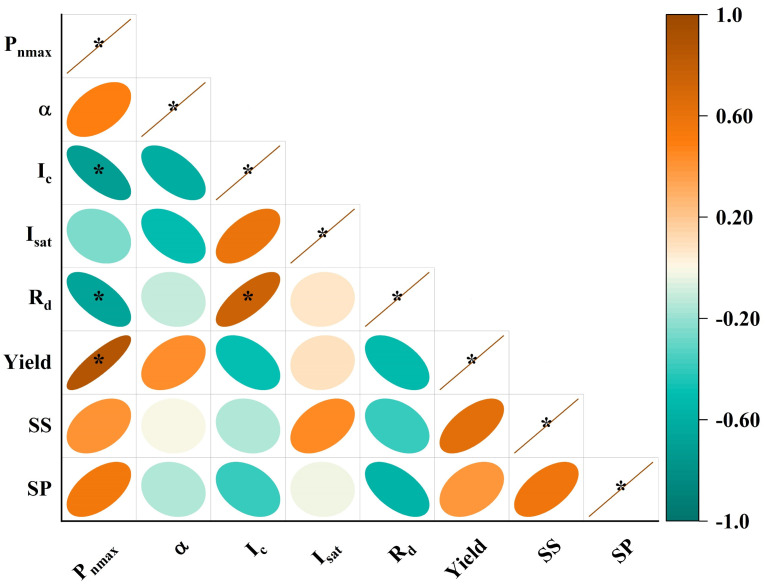
Correlation between photosynthetic characteristics parameters and yield and quality of pakchoi. * indicates a significant correlation at *p* < 0.05. P_nmax_, maximum net photosynthesis rate; α, apparent quantum efficiency; I_c_, light compensation point; I_sat_, light saturation point; R_d_, dark respiration rate; SS, soluble sugar; SP, soluble protein.

Figure 5 illustrates the correlations between pakchoi photosynthetic parameters and its yield, SS, and SP levels. There was a significant negative correlation between P_nmax_ and both the I_c_ (*r* = −0.705, *p* < 0.05) and R_d_ (*r* = −0.666, *p* < 0.05), while a significant positive correlation exists between P_nmax_ and yield (*r* = 0.863, *p* < 0.05). Although not statistically significant, P_nmax_ positively correlates with both SS and SP, underscoring the influence of photosynthesis on pakchoi quality in production. Importantly, an increase in R_d_ is negatively correlated with yield, SS, and SP, suggesting that higher R_d_ levels boost leaf physiological activity and expedite the consumption of organics, leading to a decrease in pakchoi yield and quality.

**Figure 6:**
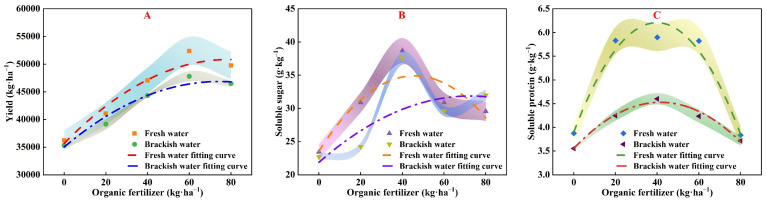
Relationship between yield (**A**), soluble sugar (**B**), soluble protein (**C**) and organic fertilizer under fresh water and brackish water irrigation.

Figure 6 and Table 4 reveal that pakchoi yield (Figure 6A), SS (Figure 6B) and SP (Figure 6C) initially increase and then decrease as the application of organic fertilizer rises. Under fresh-water irrigation, optimal values for yield, SS, and SP were obtained at organic fertilizer application rates of 74.63, 45.33, and 40.79 kg/ha, reaching peaks of 50,832 kg/ha for yield, 35.63 g/kg for SS, and 6.25 g/kg for SP, respectively. Under brackish water irrigation, optimal values for yield, SS, and SP were obtained at organic fertilizer application rates of 76.18, 56.91, 40.79 kg/ha, reaching peaks of 47,127 kg/ha for yield, 32.97 g/kg for SS, and 4.46 g/kg for SP, respectively. This pattern indicates that achieving optimal yield and quality in brackish water conditions requires a higher application of organic fertilizer [29,31].

## 4. Discussion

### 4.1. Effects of Irrigation and Fertilization on Photosynthetic Characteristics and Yield of Pakchoi

This study examines the effects of organic fertilizer on the photosynthetic attributes and yield of pakchoi under the conditions of both fresh water and brackish water irrigation. The P_n_ serves as a crucial metric for assessing plant health and productivity, signifying the plant capacity to synthesize organic compounds from atmospheric CO_2_, which is essential for growth. With increasing I, there was an initial rapid increase in the P_n_ of pakchoi, which then plateaued, demonstrating a pronounced response at low light intensities and a saturation point at higher ones. The T_r_ similarly rose with increasing I, a trend possibly associated with the elevation in G_s_, which facilitates both water transpiration and CO_2_ absorption [35,36]. Notably, C_i_ decreased with rising I, particularly at lower light levels, likely due to enhanced photosynthetic activity consuming more CO_2_ [37]. The enhanced photosynthetic parameters observed with fresh-water irrigation, such as increased P_n_, T_r_ and G_s_, may result from the lack of salt stress. Fresh water provides an optimal environment for both photosynthesis and transpiration, as salt stress can inhibit plant water uptake and cause ionic imbalances, thereby reducing photosynthetic characteristics. Although brackish water serves as a viable alternative where fresh water is limited, it is essential to manage its use carefully to prevent adverse salt effects on plant growth.

Differential responses in P_n_, T_r_, G_s_, and C_i_ across treatments suggest that organic fertilizer influences pakchoi photosynthetic efficiency. Parameters in treatments lacking organic fertilizer were consistently lower compared to those with organic fertilizer, indicating that its application enhances pakchoi photosynthesis and water use efficiency. However, the variance observed amongst fertilization levels implies that an excess of organic fertilizer does not correlate with incremental benefits, as the yield gains from photosynthesis tend to level off beyond a certain fertilization threshold. For example, P_n_ peaked with moderate organic fertilizer applications and then declined. Specifically, the highest P_n_ was detected at 60 kg/ha of organic fertilizer, especially under slightly saline irrigation, hinting at an improvement in pakchoi physiological resilience to salinity, potentially due to the presence of nutrients or biostimulants in the organic fertilizer that alleviate the negative impacts of salinity on the photosynthetic mechanism [38]. Similar to the conclusion of the previous study, our study corroborates that organic fertilizers can substantially elevate photosynthesis rates and that the high organic matter content within can improve yield [39,40]. This finding is pivotal given the extensive criticism by researchers of unsustainable agricultural practices that rely heavily on chemical fertilizers for increased yield at the expense of environmental quality and long-term agricultural viability [41,42,43]. However, it is important to note that the promotional effects of organic fertilizer on photosynthesis and yield exhibit a threshold [17].

The study further reveals that pakchoi yield, SS, and SP content initially increase with the addition of organic fertilizer but begin to decrease when application rates exceed a certain limit (Figure 6). This trend is likely due to the amelioration of soil fertility and nutrient availability up to a point, beyond which excess fertilizer may result in nutrient oversupply, negatively impacting plant growth [44,45]. The relationship delineated by fitting curves under different irrigation conditions underscores the nuanced impact of organic fertilizer on crop photosynthesis and yield. For instance, under fresh-water irrigation, the optimum yield was achieved at a fertilizer rate of 74.63 kg/ha, while the best quality of pakchoi necessitated different rates, as depicted in our models. Furthermore, the content variation in SS, a crucial source for energy and carbon skeletons, reflects the efficacy of nutrient uptake and utilization by plants [46]. Notably, under brackish water irrigation conditions, the peak values for SS and SP were attenuated compared to those under fresh water, possibly due to the influence of salinity on photosynthetic, carbohydrate, and nitrogen metabolism [47].

### 4.2. Characteristics of Light Response Model

The light response curve is a pivotal tool for gauging a plant photosynthetic response to varied light intensities and is integral to assessing photosynthetic capacity [48]. The curve fit varies according to the distinct characteristics of each model [49]. Therefore, model selection for light response analysis should align with the specific environmental conditions in which the crop is cultivated [50,51]. This study employed the following four established models to approximate the light response curve of pakchoi: The rectangular hyperbolic, non-rectangular hyperbolic, exponential, and modified rectangular hyperbolic models. The non-rectangular hyperbolic model demonstrated the least congruence with the empirical data, while the modified rectangular hyperbolic model, providing the most accurate fit, was deemed optimal for representing the light response of organically fertilized pakchoi (Figure 3, Table 2). This result infers that photosynthetic performance may be considerably impacted by non-linear dynamics under specific lighting conditions.

Table 3 reveals the effects of varying organic fertilizer applications on pakchoi photosynthetic parameters as delineated by the modified rectangular hyperbolic model. As fertilizer application increased, both the P_nmax_ and the ∆I first increase and then decrease, with the optimum P_nmax_ observed in the F3 treatment and the maximum ∆I in the F1 and B2 treatments. The apexes for P_nmax_ manifesting at a precise fertilizer threshold (Figure 4), indicate that a judicious quantity of organic fertilizer can considerably enhance photosynthetic capacity. The inverse relationship between the a and I_c_ implies that organic fertilizer boosts photosynthetic efficiency, particularly under low light. Post-fertilization decreases in the I_sat_ and increases in I_c_ suggest that excessive light might diminish photosynthetic potential, likely linked to the augmented soil fertility that organic fertilizer provides, which encourages growth in low light conditions [52]. The R_d_ indicates the utilization of photosynthetic products, with a lower rate signifying enhanced plant adaptability to stress [53,54]. The significant disparities in the R_d_ across treatments are noteworthy, with F4 and B4 exhibiting heightened R_d_, indicative of more vigorous nocturnal respiration and energy usage. Conversely, the F3 treatment lowest R_d_ intimates a more effective use of organic matter to promote growth. The application of organic fertilizer under fresh-water irrigation substantially increased the P_nmax_ in pakchoi, indicating that organic fertilizers may supplement essential nutrients to enhance the plant photosynthesis. Conversely, although organic fertilizer expanded the range of light intensities utilizable by pakchoi in brackish water irrigation conditions, it may also have hastened the consumption of organic substances and nutrients. Hence, although organic fertilizer offers direct agronomic advantages, their use in brackish water irrigation could result in a more rapid consumption of vital nutrients, necessitating a balanced approach to maintain long-term crop growth and sustainable resource utilization.

## 5. Conclusions

This investigation assesses the influence of organic fertilizer on the photosynthetic traits, yield, and quality of pakchoi irrigated with both fresh water and brackish water. The results demonstrate that a judicious application of organic fertilizer can significantly improve the photosynthetic efficiency, yield, and quality of pakchoi. Among various models, the modified rectangular hyperbolic model emerged as the most fitting for characterizing the photosynthetic response of pakchoi to organic fertilization. Key parameters, such as P_nmax_, ∆I, yield, SS, and SP, displayed a downward-opening quadratic parabolic trend with increasing levels of organic fertilizer. This study has pinpointed the optimal organic fertilizer rate to achieve the peak of P_nmax_, yield, and quality in pakchoi. The interplay between irrigation water quality and fertilizer quantity significantly influences crop yield and quality and must be duly considered in agricultural practices. Future research should delve deeper into the dynamics between water salinity stress and nutrient management, unraveling the intricate relationships between rates of organic fertilizer application and the physiological growth of crops, with the aim of devising more empirically grounded and sustainable approaches to agricultural development.

## Figures and Tables

**Table 1:** Initial physical and chemical properties of soil.

Depth (cm)	Bulk Density (g·cm^−3^)	Mechanical Composition (%)			Alkali-Hydrolyzed Nitrogen (mg·kg^−1^)	Available Phosphorus (mg·kg^−1^)	Available Potassium (mg·kg^−1^)	Organic Matter (g·kg^−1^)
		Clay	Silt	Sand				
0–20	1.45	5.3	52.5	42.2	24.34	41.37	240	8.10
20–40	1.37	3.3	32.9	63.8	13.83	8.79	144	5.10

**Table 2:** Comparison and analysis of the simulation accuracy of the light response curve by four models under different treatments.

Treatments	Rectangular Hyperbolic Model			Non-Rectangular Hyperbolic Model			Modified Rectangular Hyperbolic Model			Exponential Model		
	*R* ^2^	RMSE	RE	*R* ^2^	RMSE	RE	*R* ^2^	RMSE	RE	*R* ^2^	RMSE	RE
F0	0.983	0.6282	0.0044	0.999	2.4456	0.0664	0.990	0.4770	0.0025	0.996	0.2861	0.0009
F1	0.987	0.5779	0.0028	0.999	2.6259	0.0577	0.994	0.4048	0.0014	0.997	0.2918	0.0007
F2	0.986	0.7995	0.0027	0.996	5.8056	0.1417	0.994	0.5344	0.0012	0.996	0.4148	0.0007
F3	0.979	1.1974	0.0039	0.999	2.4317	0.0159	0.993	0.7774	0.0016	0.996	0.4813	0.0006
F4	0.992	0.6182	0.0010	0.998	9.3961	0.2388	0.995	0.4745	0.0006	0.995	1.8696	0.0095
B0	0.987	0.3915	0.0031	0.999	2.3158	0.1073	0.994	0.2555	0.0013	0.998	0.1385	0.0004
B1	0.984	0.6101	0.0049	0.999	2.3278	0.0715	0.992	0.4467	0.0026	0.997	0.2844	0.0011
B2	0.993	0.4001	0.0023	0.998	5.3386	0.4135	0.996	0.2968	0.0013	0.997	0.2724	0.0011
B3	0.987	0.7684	0.0037	0.998	2.1734	0.0292	0.994	0.5161	0.0016	0.995	0.4974	0.0015
B4	0.990	0.5241	0.0022	0.995	7.3740	0.4320	0.995	0.3769	0.0011	0.994	0.3958	0.0012

F0, F1, F2, F3, and F4 denote the application rates of organic fertilizer at 0, 20, 40, 60, and 80 kg/ha, respectively, under fresh-water irrigation. B0, B1, B2, B3, and B4 denote the application rates of organic fertilizer at 0, 20, 40, 60, and 80 kg/ha, respectively, under brackish water irrigation.

**Table 3:** Parameters of modified rectangular hyperbolic model light response curve of pakchoi under different treatments.

Treatments	Maximum Net Photosynthesis Rate (µmol·m^−2^·s^−1^)	Apparent Quantum Efficiency	Light Compensation Point (µmol·m^−2^·s^−1^)	Light Saturation Point (µmol·m^−2^·s^−1^)	Dark Respiration Rate (µmol·m^−2^·s^−1^)	∆I (µmol·m^−2^·s^−1^)	*R* ^2^
F0	11.46 de ± 0.56	0.0858 bc ± 0.0042	52.63 d ± 0.0066	1606 i ± 0.24	3.65 ab ± 0.18	1553 i ± 0.103	0.990
F1	13.58 cd ± 0.67	0.0493 de ± 0.0024	26.59 g ± 0.0007	1850 c ± 0.08	1.23 ef ± 0.06	1824 c ± 0.041	0.993
F2	18.77 b ± 0.92	0.0833 bc ± 0.0041	17.80 h ± 0.0001	1698 e ± 0.03	1.41 def ± 0.07	1679 f ± 0.015	0.994
F3	23.38 a ± 1.15	0.0999 bc ± 0.0049	10.70 i ± 0.4512	1539 j ± 0.05	1.03 f ± 0.01	1527 j ± 0.018	0.992
F4	19.14 ab ± 1.09	0.1995 a ± 0.0098	10.22 j ± 0.3141	1690 f ± 0.01	1.89 de ± 0.04	1686 e ± 0.003	0.995
B0	8.62 e ± 0.42	0.0500 de ± 0.0025	45.76 e ± 0.0014	1667 h ± 0.01	1.96 d ± 0.10	1622 g ± 0.002	0.994
B1	10.69 de ± 0.52	0.0687 cd ± 0.0034	77.95 b ± 0.0054	1685 g ± 0.28	4.20 a ± 0.21	1608 h ± 0.128	0.991
B2	10.68 de ± 0.52	0.0414 e ± 0.0020	89.45 a ± 0.0110	2184 a ± 0.13	3.10 bc ± 0.15	2094 a ± 0.072	0.996
B3	16.93 bc ± 0.83	0.0321 e ± 0.0016	60.20 c ± 0.0003	2086 b ± 0.01	1.83 de ± 0.09	2026 b ± 0.005	0.994
B4	12.54 cde ± 0.66	0.0888 bc ± 0.0044	35.10 f ± 0.0008	1740 d ± 0.02	2.70 c ± 0.13	1705 d ± 0.009	0.995

∆I = I_sat_ − I_c_, I_sat_, light saturation point, I_c_, light compensation point. F0, F1, F2, F3, and F4 denote the application rates of organic fertilizer at 0, 20, 40, 60, and 80 kg/ha, respectively, under fresh-water irrigation. B0, B1, B2, B3, and B4 denote the application rates of organic fertilizer at 0, 20, 40, 60, and 80 kg/ha, respectively, under brackish water irrigation. Different lowercase letters indicate significant differences at *p* < 0.05. The value is shown as the means ± SD (n = 3).

**Table 4:** Mathematical models of yield, soluble sugar (SS), soluble protein (SP), and organic fertilizer (OF) under fresh water and brackish water irrigation.

Irrigation Water	Mathematical Model	*R* ^2^	*p*
Fresh water	Yield = −2.7732 × OF + 413.92 × OF + 35387	0.950	<0.05
	SS = −0.0059 × OF + 0.5349 × OF + 23.503	0.793	
	SP = −0.0014 × OF + 0.1142 × OF + 3.9225	0.963	
Brackish water	Yield = −2.1405 × OF + 326.11 × OF + 34706	0.966	
	SS = −0.0035 × OF + 0.3984 × OF + 21.632	0.585	
	SP = −0.0006 × OF + 0.0466 × OF + 3.5578	0.978	

## Data Availability

Data are contained within the article.
